# Insights into Mesalazine Use in Clinical Practice of Young Gastroenterologists

**DOI:** 10.3390/jcm12052005

**Published:** 2023-03-02

**Authors:** Olga Maria Nardone, Giovanni Marasco, Loris Riccardo Lopetuso, Giammarco Mocci, Luca Pastorelli, Carlo Petruzzellis, Franco Scaldaferri, on behalf of the Italian Association of Young Gastroenterologist and Endoscopist (AGGEI)

**Affiliations:** 1 Gastroenterology, Department of Public Health, University Federico II of Naples, 80131 Naples, Italy; 2 Division of Internal Medicine and Digestive Physiopathology, IRCCS Azienda Ospedaliero-Universitaria di Bologna, Policlinico S. Orsola, 40138 Bologna, Italy; 3 Department of Medical and Surgical Sciences, University of Bologna, 40138 Bologna, Italy; 4 CEMAD—IBD UNIT—Unità Operativa Complessa di Medicina Interna e Gastroenterologia, Dipartimento di Scienze Gastroenterologiche, Endocrino-Metaboliche e Nefro-Urologiche, Fondazione Policlinico Universitario “A. Gemelli” IRCCS, 00168 Rome, Italy; 5 Division of Gastroenterology, “Brotzu” Hospital, 09124 Cagliari, Italy; 6 Gastroenterology and Hepatology Unit, ASST Santi Paolo e Carlo, 20142 Milan, Italy; 7 Department of Health Sciences, University of Milan, 20142 Milan, Italy; 8 Gastroenterology Unit, Azienda Ospedaliera di Rilievo Nazionale e di Alta Specializzazione Garibaldi, 95123 Catania, Italy

**Keywords:** mesalazine, inflammatory bowel disease, ulcerative colitis, survey, training, educational program

## Abstract

Background: Mesalazine is among the medications most prescribed by gastroenterologists, with variable and controversial use in different settings. We aimed to explore the use of mesalazine in the clinical practice of young gastroenterologists. Methods: A web-based electronic survey was distributed to all participants of the National Meeting of the Italian Young Gastroenterologist and Endoscopist Association. Results: A total of 101 participants took part in the survey, with a majority (54.4%) being aged >30 years, 63.4% of whom were trainees in academic hospitals, and 69.3% of whom were involved in the clinical management of inflammatory bowel disease (IBD). While both non-dedicated and IBD physicians generally agreed on the appropriate dose of mesalazine for mild ulcerative colitis (UC), significant differences were observed between the two groups for moderate-severe ulcerative colitis (UC). Additionally, in IBD patients who were starting immuno-modulators and/or biologics, 80% of IBD-dedicated physicians continued to prescribe mesalazine, compared to 45.2% of non-dedicated physicians (*p* = 0.002). Indeed, 48.4% of non-dedicated IBD physicians did not acknowledge mesalazine for colorectal cancer chemoprevention. With regards to Crohn’s disease, it is mainly used by 30.1% of IBD physicians for preventing postoperative recurrence of Crohn’s disease. Finally, 57.4% used mesalazine for symptomatic uncomplicated diverticular disease, and 84.2% did not recommend its use for irritable bowel syndrome. Conclusions: This survey showed heterogeneous behaviors in the daily use of mesalazine, mainly in the management of IBD. Educational programs and novel studies are needed to clarify its use.

## 1. Introduction

Mesalazine, also known as 5-aminosalicylic acid (5-ASA), is an anti-inflammatory drug used to treat inflammatory bowel diseases (IBD) and other inflammatory diseases of the digestive tract. For more than thirty years, aminosalicylates (5-ASA) have remained the mainstay of therapeutic strategies in ulcerative colitis (UC) patients [1]. Indeed, a systematic review by Fumery has shown that over 90% of patients receive a 5-ASA within the first year of diagnosis, with between 60% and 90% continuing their use up to 15 years [2]. However, in recent years, the drug pipeline in IBD has become richer than ever before, and promising new treatments are going to be integrated alongside our existing therapeutics [3]. Therefore, profiling patients with risk stratification and choosing the right therapy for the right person at the right time represents the basis for IBD management [4]. The current guidelines recommended the use of 5-ASA for the induction and maintenance of remission in patients with mild to moderate UC [5]. While, positioning 5-ASA for patients with moderate-to-severe UC is challenging, especially in the context of biological therapies. For Crohn’s disease (CD), international guidelines do not recommend the use of 5-ASA except in some specific clinical situations, such as the postoperative prophylactic treatment among patients at low risk of relapse [5].

Mesalazine use was also explored in other inflammatory conditions of the digestive tract, such as the treatment of acute diverticulitis or symptomatic uncomplicated diverticular disease (SUDD) [6,7,8,9] and irritable bowel syndrome (IBS) [10,11], although with no conclusive results. Therefore, there are still grey areas to explore, and noteworthy, several beliefs and attitudes are in sharp contrast to current guidelines. In order to explore the perceived role of mesalazine in the management of IBD and other inflammatory conditions of the digestive tract in the era of biologics and small molecules, a survey was developed by five members of the Young Italian Gastroenterologist and Endoscopist Association (Associazione Giovani Gastroenterologi ed Endoscopisti Italiani—AGGEI) Governing Board. Hence, we aimed to investigate how young gastroenterologists placed the use of mesalazine in daily clinical practice.

## 2. Methods

### 2.1. Study Design

A prospective web-based survey investigating the current use of mesalazine in clinical practice by young gastroenterologists and GI trainees was developed through a videoconference meeting by a task force including 5 members representative of the AGGEI. The final questionnaire of the survey was internally validated and finally approved by all the members of the AGGEI Steering Committee.

The survey was conducted in accordance with the World Medical Association Declaration of Helsinki. Written informed consent was obtained from participants to participate in the study.

### 2.2. Development and Content of the Questionnaire

The survey was in the Italian language, and participants answered based on what they observed being utilized in their patients’ daily clinics. It involved the main setting wherein mesalazine is used regardless of mesalazine formulation. In detail, it consisted of a brief introduction of the project and 3 sections, which included a total of 28 multiple-choice questions. The following areas of interest were explored: (a) demographics and work position baseline, (b) use of mesalazine in the management of IBD, and (c) use of mesalazine in other gastrointestinal diseases such as diverticular disease and irritable bowel syndrome. Full survey questions and responses are available in tables below.

### 2.3. Distribution of Questionnaire and Collection of Data

The electronic version of the survey was distributed via e-mail to all the AGGEI members participating in the annual National Meeting of the association. All subjects accepted to participate in the survey through informed consent for the handling and collection of data for scientific purposes. The survey was conducted between 12 November 2021 and 28 February 2022.

### 2.4. Statistical Analysis

Data are presented as counts and percentages for the categorical variables and mean and standard deviation (SD) for the continuous variables. The categorical variables were compared using the Chi-squared or Fisher’s exact tests as appropriate. For multiple categorical variables, the Chi-squared test of independence was used. The continuous variables were compared using the t-test or the Kruskal–Wallis test as appropriate. The differences in responses between physicians routinely taking care of IBD (IBD physicians) and not daily involved in IBD management (non-dedicated IBD physicians) were calculated. The probability values were two-sided; a probability value of less than 0.05 was considered statistically significant. Statistical analysis was performed with STATA 17.0 (SE, Standard Edition, College Station, TX: StataCorp LP).

## 3. Results

### 3.1. Demographics and Professional Data

Among all trainees in gastroenterology and/or young gastroenterologists invited, 101 completed the survey (101/241, 41.9%). Half of the participants involved in the survey were males (52/101, 51.5%). More than half of the participants (54.4%) were aged >30 years (30–35 years 38/101, 37.6%, >35 years 17/101, 16.8%). The majority came from North-East Italy (Friuli-Venezia Giulia, Veneto, Emilia Romagna, Province of Trento/Bolzano) (42/101, 31.7%). Most of the participants worked in academic hospitals (64/101, 63.4%), given that the majority of them were gastroenterologists still in training (53, 52.5%). Demographics and professional data are detailed in Table 1.

### 3.2. Mesalazine Use in Inflammatory Bowel Diseases

In total, 70 out of 101 participants (69.3%) reported being involved in the clinical management of IBD, as shown in Table 2, which presents the full report of questionnaire responses. In comparison to non-dedicated IBD physicians, a significantly higher percentage of IBD physicians reported knowledge of three formulations of mesalazine (51.6% vs. 92.9%, *p* < 0.001). Regarding the doses of mesalazine for the treatment of mild UC, no significant differences were observed between non-dedicated IBD and IBD physicians (*p* = 0.062). While, significant differences were found between non-dedicated IBD vs. IBD physicians for the treatment of moderate-severe UC, with 38.7% and 62.9%, respectively, using the maximum dosage of 4000–4800 mg/day (*p* = 0.012). No significant differences were found in the treatment of active ulcerative proctitis, and most participants used a combination of topical and oral mesalazine. Additionally, no differences were found in the length of combination therapy with topical and oral mesalazine in left-sided colitis, with the majority reporting a 6-week course (41.9% non-dedicated IBD vs. 30% IBD) or continuing until the achievement of remission (35.3% non-dedicated IBD vs. 42.9% IBD, *p* = 0.391). Almost all non-dedicated IBD and IBD physicians advocated for the use of mesalazine for the maintenance of UC regardless of other therapies (*p* = 0.144). Similarly, no significant differences were reported regarding the doses of mesalazine for maintenance of UC, with more than 70% of respondents in both groups reporting the use of 2400 mg/day (*p* = 0.586).

Another important aspect of the study was to assess whether physicians continued prescribing mesalazine in patients who started on immunomodulators and/or biologics. The results showed that 80% of IBD-dedicated physicians continue both therapies compared to only 45.2% of non-dedicated IBD physicians (*p* = 0.002). Furthermore, in patients with inadequate response to mesalazine who require a second-line therapy, 32.3% of non-dedicated IBD physicians discontinue mesalazine, compared to only 7% of IBD dedicated (*p* = 0.007).

Heterogeneous behaviors were reported for CD, with 25.8% of non-IBD physicians and 30.1% of IBD physicians using mesalazine for postoperative recurrence without statistical significance (*p* = 0.866).

### 3.3. Administration, Safety, Adherence, and Prevention in Inflammatory Bowel Diseases

Significant differences were found in the maximum dose titration for UC among non-dedicated IBD and IBD physicians, although the majority of both groups reported using a dosage of up to 4800 mg/day (67.7% vs. 90%, respectively, *p* = 0.026). Most IBD and non-dedicated IBD physicians reported that there are no evidence-based time limits for the administration of mesalazine at the maximum dose, although a non-negligible rate of non-dedicated IBD reported advising maximum dose for the shortest time possible (*p* = 0.006). No significant difference was found in the suggestion for improving adherence to mesalazine therapy (*p* = 0.21), even if most IBD participants suggested single daily dosing, while non-dedicated IBD participants had therapeutical education. Interestingly, more than half of respondents of both groups referred to differences in efficacy and safety among the different mesalazine formulations. The most frequent adverse events reported were nausea (56.4%) and headache (32.7%), while the majority of participants reported checking renal function every 6–12 months (68.3%). Heterogeneous behaviours were found in chemo-preventive effect against colorectal cancer since most IBD physicians (45.7%) reported the use of 2000–2400 mg/day, while 48.4% of non-dedicated IBD physicians have never used it (*p* = 0.040). With regards to prophylaxis with topical mesalazine in patients with proctitis, most non-dedicated IBD physicians reported avoiding prophylaxis (45.2%), while the majority of IBD physicians advised mesalazine twice a week (42.9%, *p* = 0.203) (Table 3).

### 3.4. Mesalazine Use in Other Chronic Gastrointestinal Diseases

More than half of the participants (57.4%) in this survey reported using mesalazine for the treatment of symptomatic uncomplicated diverticular disease, most of them for 7 or 10 days per month (71.8%). However, most physicians (66.3%) interviewed reported not using mesalazine for the prevention of acute diverticulitis recurrence or in the treatment of patients with irritable bowel syndrome (IBS) (Table 4).

## 4. Discussion

This survey provides a snapshot of the current thinking and decision-making of young Italian gastroenterologists about the use of mesalazine, especially in the therapeutic algorithms of IBD. Our results reveal an adequate knowledge of guidelines and a quite homogenous behavior among all interviewed gastroenterologists. A greater agreement in therapeutic behavior was achieved for the treatment of mild diseases. These results should be positively interpreted given the recent enrichment of the drug pipeline for IBD [3], which sometimes may be confusing, especially for not dedicated physicians, leading to a risk of overtreatment. Hence, this witnesses that today there is still a role for positioning mesalazine in the algorithm of mild UC. Conversely, we observed significant discrepancies in the treatment of moderate-severe UC between IBD and non-dedicated IBD physicians. These could be explained by the lack of definition of moderate UC, which is still considered a grey area and results in heterogeneous clinical practice with the risk of under or over-treatment.

To date, few studies have examined how patients with UC are treated by several physicians in clinical practices. In 2010, a survey of Spanish gastroenterologists reported a high degree of agreement with European guidelines in the management of mild to moderate UC between general gastroenterologists and those specialized in IBD. However, less agreement was observed in the general gastroenterologist group, in which increased maintenance treatment with mesalazine, the use of a single daily dose of mesalazine, and the use of combined oral and topical treatment for distal colitis should be promoted [12]. Similarly, an Italian prospective cross-sectional study including IBD-specialized gastroenterologists and general gastroenterologists working in Italian public hospital units reported a higher prescription of rectal and combination therapy in mild to moderate distal disease and a higher rate of hospitalization in severe UC among IBD gastroenterologists than general gastroenterologists [13]. The results from these authors confirm the huge heterogeneity defining this group of patients and emphasize that scientific societies need to work together to allow harmonization on the definition of moderately active UC.

Another worthy aspect of the survey is treatment adherence since previous studies showed that up to 60% of patients with UC treated by mesalazine are non-adherent with conventional multi-dose regimens. Here, we did not find significant differences in terms of suggestions and advice for improving adherence to mesalazine therapy, even if most IBD-specialized physicians suggested single daily dosing, while non-dedicated IBD gastroenterologists proposed therapeutical education. 

A recent multinational survey [14] involving experts in IBD investigated the clinical decision-making for the management of mild-to-moderate UC, and notably, they found that the optimization of mesalazine dosage was a key point for assuring long-term support for the patient. Accordingly, they suggested that the current management for mild-to-moderate UC should be guided by the patient’s perspectives and goals, as well as the assessment of their medical and disease history.

Regardless, many studies have reported that any medication that successfully controls inflammation and maintains remission would reduce the risk of colorectal cancer. Thus, a further benefit of the long-term use of mesalazine is the potential chemo-preventive effect. In this context, whether mesalazine should be continued in combination with thiopurines or biological therapy remains a topic of intense debate. We found a significant difference since the majority of IBD physicians continued both therapies (80%), whereas non-dedicated IBD physicians only in 45.2% of cases. Most IBD physicians (45.7%) reported the use of 2000–2400 mg/day of mesalazine for chemo-preventive purposes; surprisingly, overall, 48.4% of non-dedicated IBD physicians have never used it. Hence, in the next future, it is worth covering these aspects, which are critical in this type of long-standing progressive disease with a relapsing-remitting course.

We further explored the use of mesalazine in CD patients since there is a sharp contrast between current guidelines and clinical practice. Several studies indicated that mesalazine is no more effective than the placebo for induction and maintenance of remission in CD [15,16,17,18]. Nevertheless, it has a practical benefit, and as such, it is still prescribed by many physicians [19]. In a recent population-based cohort, more than half of CD patients received mesalazine at some point in their disease course [20]. The Epi-IBD study [21], which involved a European community-based inception cohort, reported that mesalazine was commonly used in CD, and a substantial group of these patients experienced a quiescent disease course without the need for additional treatment during follow-up. Similarly, based on our results, more than 70% of both IBD and non-dedicated IBD physicians use mesalazine to prevent clinical recurrence in CD.

Concerning safety, there were no significant differences between the two analyzed groups. Of note, despite an overall safety profile similar to a placebo, many cases of nephrotoxicity have been reported with 5-ASA. In accordance with these, most of the participants in the Survey reported monitoring kidney function to prevent injuries [22]. We did not focus on timing for monitoring; however, the monitored consensus proposed an evaluation of 3 months after starting mesalazine [23]. 

Furthermore, heterogeneous behaviours were found for the use of mesalazine in other inflammatory conditions, where few and weak data are available and unmet therapeutical needs are still present. Focusing on other chronic inflammatory gastrointestinal diseases such as diverticular disease and IBS, the use of mesalazine becomes more transversely heterogeneous in both groups. More than half of participants (57.4%) among both groups reported using mesalazine for the treatment of SUDD, most of them for 7 or 10 days per month (71.8%). In diverticular disease, mesalazine might exert anti-inflammatory activity, thereby improving chronic, low-grade inflammation and modulating the nociception [7,8,9,24]. In patients with SUDD, one double-blind study showed its efficacy in providing pain relief during symptomatic flares [25], whereas another study found that mesalazine was more effective in maintaining remission than placebo [26]. Some benefits in symptom relief in SUDD patients have been claimed by a recent metanalysis [27] reporting the effectiveness of mesalazine for the treatment of SUDD and in the prevention of the occurrence of acute diverticulitis, although supported by very few studies. On the other hand, stronger data advise against the use of mesalazine for the prevention of acute diverticulitis recurrence [28]. In the present Survey, we found that more than half of respondents used mesalazine for SUDD treatment, therefore mirroring the findings of a recent real-life Italian cohort study exploring the drugs used for the treatment of diverticular disease and reporting that mesalazine was the third most prescribed drug in this setting [29], although without strong evidence. As for IBS, most of the participants in the survey denied the use of mesalazine, according to well-designed trials [30], a recent metanalysis [31], and the recent guidelines of joint societies [32,33] which advise against the use of mesalazine in IBS.

This survey has several strengths: it provides a cutting-edge snapshot of the current knowledge and use of mesalazine among young gastroenterologists in common gastrointestinal diseases. Second, in accordance with the literature data and given the complexity of these diseases, we showed that involvement in the IBD field makes a difference in the management of IBD patients; this evidence emphasizes the importance of a dedicated and specialized IBD group to ensure high-quality care to all patients.

However, there are also some limits: it should be recognized that respondents to the questionnaire were mostly practicing in academic hospitals, with a possible bias in the description of the real-life situation on the knowledge and strategies of the utilization of mesalazine. Second, it was not possible to use validated questionnaires exploring the adherence to mesalazine as these are referred to patients. Furthermore, some heterogeneity in our results may be influenced by the different global and IBD-dedicated training levels of respondents, which was not deeply explored in this study. Regardless, the purpose of this survey was to capture the use of mesalazine among young gastroenterologists and identify areas where knowledge gaps exist. 

## 5. Conclusions

In conclusion, this survey highlights that the definition of disease activity in IBD patients is crucial for establishing the best therapeutic strategies. In this context, mesalazine can still be considered “an ace up the sleeve” of gastroenterologists in several different contexts.

However, there are still some significant discrepancies to be aware especially in moderate-severe UC, in terms of doses and the concomitant use with second-line therapy. 

Considering that this survey includes mostly trainees, it is of paramount importance to standardize the knowledge on the use of mesalazine in all clinical settings. We strongly believe that all young gastroenterologists should have adequate knowledge of basic therapy for patients, without any differences between specialized and non-specialized centers. Hence, we hope to provide a basis for further research and promote new educational programs to make the use of mesalazine more homogeneous.

## Figures and Tables

**Table 1:** Demographic data of trainees in gastroenterology and/or young gastroenterologists accepting to participate in the survey.

	Trainees in Gastroenterology and/or Young Gastroenterologists n. 101, n (%)
**Age**	
≤30	46 (45.5)
>30 and ≤35	38 (37.6)
≥35	17 (16.8)
**Gender (Male)**	52 (51.5)
**Workplace**	
North-West	21 (20.8)
North-East	32 (31.7)
Center	24 (23.8)
South and Islands	24 (23.8)
**Institution**	
Academic	64 (63.4)
Not Academic	31 (30.7)
Private hospital/practice	4 (4)
Other	2 (2)
**Clinical role**	
Trainee	53 (52.5)
PhD	11 (10.9)
Consultant	33 (32.7)
Other	4 (4)

n.: number; PhD: Doctor of Philosophy; North-West: Valle d’Aosta, Piemonte, Lombardia, Liguria; North-East: Friuli-Venezia Giulia, Veneto, Emilia-Romagna, Province of Trento/Bolzano; Center: Toscana, Marche, Umbria, Lazio; South and Islands: Abruzzo, Molise, Campania, Puglia, Basilicata, Calabria, Sicilia, Sardegna.

**Table 2:** Management of inflammatory bowel diseases with mesalazine.

	Total n (%) *n* = 101	Non-Dedicated IBD Physician n (%) *n* = 31	IBD Physician n (%) *n* = 70	*p*-Value *
**How many Mesalazine formulation do you remember?**				<0.001
1	1 (1)	1 (3.2)	0	
2	19 (18.8)	14 (45.2)	5 (7.1)	
3	81 (80.2)	16 (51.6)	65 (92.9)	
There are no different mesalazine formulations	0	0	0	
**In mild ulcerative colitis which dose of Mesalazine do you use?**				0.062
800 mg /die	5 (5)	3 (9.7)	2 (2.9)	
2000–2400 mg/die	71 (70.3)	25 (80.7)	46 (65.7)	
3000–3600 mg/die	19 (18.8)	3 (9.7)	16 (22.9)	
4000–4800 mg/die	6 (5.9)	0	6 (8.6)	
**In moderate-severe ulcerative colitis which dose of Mesalazine do you use?**				0.012
2000–2400 mg/die	4 (4)	3 (9.7)	1 (1.4)	
3000–3600 mg/die	21 (20.8)	11 (35.5)	10 (14.3)	
4000–4800 mg/ die	56 (55.5)	12 (38.7)	44 (62.9)	
I start with biological therapy or immunosuppressor	20 (19.8)	5 (16.1)	15 (21.4)	
**In active ulcerative proctitis do you generally use**				0.494
Topical mesalazine therapy alone	19 (18.8)	7 (22.6)	12 (17.1)	
Combination of topical and oral mesalazine	67 (66.3)	18 (58.1)	49 (70)	
Oral mesalazine	-	-	-	
Combining topical steroids oral mesalazine	15 (14.9)	6 (19.4)	9 (12.9)	
**How long do you use combination therapy in left sides colitis**				0.391
4 weeks	12 (11.9)	5 (16.1)	7 (10)	
6 weeks	34 (33.7)	13 (41.9)	21 (30)	
12 weeks	15 (14.9)	3 (9.7)	12 (17.1)	
till the patient achieves clinical remission				
**Do you use Mesalazine in disease maintenance in inflammatory bowel disease?**				0.144
No use for maintenance in inflammatory bowel disease	1 (1)	1 (3.2)	0	
Together with other drugs for maintenance in ulcerative colitis	34 (33.7)	8 (25.8)	26 (37.1)	
Alone for maintenance in ulcerative colitis	65 (64.4)	21 (67.7)	44 (62.9)	
Only for Crohn’s disease	1 (1)	1 (3.2)	0	
**In your clinical practice, which dose of Mesalazine do you use for ulcerative colitis mainteinance?**				0.586
1600 mg/die	9 (8.9)	4 (12.9)	5 (7.1)	
2000 mg/die	6 (5.9)	2 (6.5)	4 (5.7)	
2400 mg/die	76 (75.3)	22 (71)	54 (77.1)	
4000 mg/die	8 (7.9)	2 (6.5)	6 (8.6)	
4800 mg/die	1 (1)	0	1 (1.4)	
It is not recommended for maintenance	1 (1)	1 (3.2)	0	
**In your clinical practice, do you use Mesalazine for Crohn’s disease?**				0.866
Yes, only in Crohn’s colitis	27 (26.7)	8 (25.8)	19 (27.1)	
Yes, to prevent clinical recurrence for post-surgical Crohn’s disease	29 (28.7)	8 (25.8)	21 (30.1)	
Yes, in ileo-colonic Crohn’s disease	27 (26.7)	8 (25.8)	19 (27.1)	
No, never	18 (17.8)	7 (22.6)	11 (15.7)	
**Do you stop Mesalazine among patients starting immunomodulators and /or biologics?**				0.002
Yes	14 (13.9)	8 (25.8)	6 (8.6)	
No, I continue both therapy	70 (69.3)	14 (45.2)	56 (80)	
I continue till 6 months	17 (16.8)	9 (29)	8 (11.4)	
I continue till 12 months	0	0	0	
**During a second-line therapy for ulcerative colitis you use to:**				0.007
Withdraw mesalazine	15 (14.9)	10 (32.3)	5 (7.1)	
Reduce mesalazine dose	15 (14.9)	5 (16.1)	10 (14.3)	
Continue mesalazine at standard dose	62 (61.4)	15 (48.4)	47 (67.1)	
Increase mesalazine dose	9 (8.9)	1 (3.2)	8 (11.4)	

* *p*-value < 0.05 significant.

**Table 3:** Administration, safety, adherence, and prevention in inflammatory bowel diseases.

	Total	Non-Dedicated IBD Physician n (%) *n* = 31	IBD Physician n (%) *n* = 70	*p*-Value *
**Which is the maximum dose tritation adviced for Mesalazine in ulcerative colitis**				0.026
2400 mg/die	3 (3)	1 (3.2)	2 (2.9)	
4000 mg/die	10 (9.9)	7 (22.6)	3 (4.3)	
4800 mg/die	84 (83.2)	21 (67.7)	63 (90)	
Not available data on maximum dose	4 (4)	2 (6.5)	2 (2.9)	
**Which is the maximum time adviced for Mesalazine use at maximum dose?**				0.006
Until remission	14 (13.9)	2 (6.5)	12 (17.1)	
Shortest time possible	14 (13.9)	9 (29)	5 (7.1)	
No evidence-based time limit	63 (62.4)	15 (48.4)	48 (68.6)	
3 months	10 (9.9)	5 (16.1)	5 (7.1)	
**How do you suggest improving adherence to Mesalazine therapy**				0.210
Once daily dosing	58 (57.4)	13 (41.9)	45 (64.3)	
Therapeutic education	33 (32.7)	14 (45.2)	19 (27.1)	
New oral formulations	8 (7.9)	3 (9.7)	5 (7.1)	
Others	2 (2)	1 (3.2)	1 (1.4)	
**Do you find any differences in efficacy and safety among the different Mesalazine formulations?**				0.016
Yes	56 (55.5)	16 (51.6)	40 (57.1)	
No	19 (18.8)	2 (6.5)	17 (24.3)	
I don’t know	26 (25.7)	13 (41.9)	13 (18.6)	
**Which is the most frequent adverse event that you register in your clinical practice?**				0.042
Nasopharyngitis	3 (3)	3 (9.7)	0	
Nausea	57 (56.4)	18 (58.1)	39 (55.7)	
Headache	33 (32.7)	9 (29)	24 (34.3)	
Pancreatitis	8 (7.9)	1 (3.2)	7 (10)	
**How often do you check renal function during Mesalazine treatment?**				0.140
Never	13 (12.9)	6 (19.4)	7 (10)	
Every month	1 (1)	1 (3.2)	0	
2–3 months	18 (17.8)	7 (22.6)	11 (15.7)	
6–12 months	69 (68.3)	17 (54.8)	52 (74.3)	
**Which dose of Mesalazine do you use for chemopreventive effect against colo-rectal cancer?**				0.040
Never	35 (34.6)	15 (48.4)	20 (28.6)	
800 mg/die	12 (11.9)	6 (19.4)	6 (8.6)	
≤1200 mg/die	15 (14.9)	3 (9.7)	12 (17.1)	
2000–2400 mg/die	39 (38.6)	7 (22.6)	32 (45.7)	
**How do you manage prophylaxis with Mesalazine topical therapy in patients with proctitis?**				0.203
No prophylaxis	33 (32.7)	14 (45.2)	19 (27.1)	
10 days/month	18 (17.8)	6 (19.4)	12 (17.1)	
Twice a week	37 (36.6)	7 (22.6)	30 (42.9)	
Other	13 (12.9)	4 (12.9)	9 (12.9)	

* *p*-value < 0.05 significant.

**Table 4:** Management of other chronic gastrointestinal diseases.

	Total	Non-Dedicated IBD Physician n (%) *n* = 31	IBD Physician n (%) *n* = 70	*p*-Value *
**Do you use Mesalazine for the treatment of symptomatic uncomplicated diverticular disease (SUDD)?**				0.432
Yes	58 (57.4)	16 (51.6)	42 (60)	
No	43 (42.6)	15 (48.4)	28 (40)	
**If yes, how many days per month do you prescribe Mesalazine in SUDD patients?**				0.013
5	6 (11.3)	4 (25)	2 (5.4)	
7	19 (35.9)	8 (50)	11 (29.7)	
10	19 (35.9)	1 (6.3)	18 (48.7)	
14	9 (17)	3 (18.8)	6 (16.2)	
**Do you use Mesalazine for the prevention of acute diverticulitis recurrence?**				0.465
No	67 (66.3)	24 (77.4)	43 (61.4)	
Yes, alone	4 (4)	1 (3.2)	3 (4.3)	
Yes, in combination with rifaximin	26 (25.7)	5 (16.1)	21 (30)	
Yes, in combination with probiotics	4 (4)	1 (3.2)	3 (4.3)	
**Do you use Mesalazine in patients with irritable bowel syndrome?**				0.466
No	85 (84.2)	24 (77.4)	61 (87.1)	
Yes	7 (6.9)	3 (9.7)	4 (5.7)	
Only in IBS with diarrhea	9 (8.9)	4 (12.9)	5 (7.1)	
Only in IBS with constipation	-	-	-	

* *p*-value < 0.05 significant.

## Data Availability

All data generated or analyzed during this study are included in this article file. Further inquiries can be directed to the corresponding author.
